# To Die or to Survive, a Fatal Question for the Destiny of Prostate Cancer Cells after Androgen Deprivation Therapy

**DOI:** 10.3390/cancers3021498

**Published:** 2011-03-24

**Authors:** Kai-Xin Zhang, Jessica Firus, Brenda Prieur, William Jia, Paul S. Rennie

**Affiliations:** 1 The Vancouver Prostate Centre, 2660 Oak St., Vancouver, BC V6H 3Z6, Canada; E-Mails: Kevin.Zhang@vch.ca (K.-X.Z.); jfirus@prostatecentre.com (J.F.); bprieur@prostatecentre.com (B.P.); 2 Department of Urologic Sciences, University of British Columbia, Vancouver, BC V6H 3Z6, Canada; 3 Department of Surgery and Brain Research Centre, University of British Columbia, Vancouver, BC V6H 3Z6, Canada; E-Mail: wjia@interchange.ubc.ca

**Keywords:** prostate cancer, cell death mechanisms, apoptosis, progression, castration resistance

## Abstract

Prostate cancer is the most frequently diagnosed non-skin cancer in adult males in North America and is the second leading cause of cancer-related mortality. For locally advanced or metastatic disease, androgen deprivation, through medical or surgical castration, is the primary treatment to induce prostate cancer cell death and extend patient survival. However, the vast majority of cancers progress to a castration-resistant/androgen-independent state where the cell death processes are no longer active. This review describes the main cell death processes, apoptosis, autophagy, necrosis and necroptosis, which may be activated in prostate cancers after androgen deprivation therapy as well as the molecular mechanisms through which the cancers progress to become castration resistant. In particular, the central role of persistent androgen receptor (AR)-mediated signaling and AR crosstalk with other critical cell signaling pathways, including (i) the PI3K/Akt pathway, (ii) receptor tyrosine kinases, (iii) the p38 MAPK pathway, and (iv) the Wnt/β-catenin pathway, as well as reactivation of AR by *de novo* synthesized androgen are discussed in this context. Understanding the molecular changes that subvert normal cell death mechanisms and thereby compromise the survival of prostate cancer patients continues to be a major challenge.

## 1. Introduction

Prostate cancer is the most commonly diagnosed non-skin cancer in men and one of the leading causes of cancer-death [1]. It has been estimated that there will be almost 217,730 newly diagnosed prostate cancer cases and 32,050 of them will die of the disease this year in the United States. While frequently curable in its early stages by surgery or radiotherapy, many patients will either have a later recurrence or initially present with locally advanced or metastatic disease [2,3]. The primary treatment for advanced metastatic prostate cancer is androgen deprivation therapy (ADT). In both normal and neoplastic prostates, the male sex hormones, termed androgens, play critical roles in cell growth and survival [4]. Androgen-mediated mitogenic and survival signaling are transduced through a protein called the androgen receptor (AR), which functions as an androgen-activated transcription factor to regulate specific genes. The reliance of prostate cancer cells on androgens provides the basis for using ADT as the first line treatment for advanced prostate cancer. ADT may be achieved through surgical (*i.e.*, removal of the testes) or medical castration. The latter is usually achieved by administration of either LH-RH agonists or antagonists, which cause an inhibition of testicular androgen production. ADT may also involve combining castration with anti-androgen therapy, where steroidal or nonsteroidal AR antagonists are given to block androgen-mediated growth and survival signaling through direct competition with androgens for binding to the AR [5-7]. After ADT, cell death occurs causing involution of normal prostate glandular epithelia and regression of androgen-dependent prostate cancer cells [8]. While apoptosis is generally believed to be the primary mechanism for programmed cell death in prostate cancers, other forms of cell death may also be involved and are also discussed in this review.

## 2. Cellular Mechanisms for Cell Death

### 2.1. Apoptosis

At least four major cell death modalities (apoptosis, autophagy, necrosis and necroptosis) have been described [9-11]. Apoptosis, considered type 1 cell death, is caused by either polymerizations of death receptors such as FAS and TNF-R1 by extrinsic FAS ligand or TNF-α binding, or elicited by intrinsic signals that induce mitochondria to release cytochrome C, which acts as a potent catalyst for apoptosis. Members of the Bcl-2 super family of proteins have distinct activities, either pro-apoptotic (Bax, Bak, Bid, Bim) or anti-apoptotic (Bcl-2, Bcl-XL, Bcl-W), which regulate the apoptotic process by controlling the mitochondrial death signaling through cytochrome C release [11-13]. The redundancy of key molecules to control apoptosis may cross regulate with and compensate for each other. In both extrinsic and intrinsic signaling-mediated apoptosis, cleavage-activation of an array of intracellular proteases within cells, called caspases, leads to execution of the cell death program [14]. Morphologically, apoptosis is defined by nuclear pyknosis (chromatin condensation) and karyorhexis (nuclear fragmentation). Progressive cellular condensation and budding form apoptotic bodies surrounded by membranes and contain intact cytoplasmic organelles or fragments of the nucleus [11,13]. These apoptotic bodies are eventually engulfed by resident phagocytes. Apoptosis is involved in tissue homeostasis, embryonic development, and disease initiation and progression.

### 2.2. Autophagic Cell Death

While apoptosis may induce rapid destruction of all cell structures and organelles, type 2 cell death, referred to as autophagic cell death, involves a rather slow process [11]. Upon nutrient deprivation, growth factor withdrawal or other stressors, autophagosomes, which are double-membrane vacuoles, are formed in eukaryotic cells [11]. Autophagosomes sequester cytoplasmic proteins, mitochondria, endoplasmic reticulum, and ribosomes for eventual degradation by lysosomal hydrolases. This “self-eating” process called autophagy is a means of disposing of defective organelles and macromolecular structures, as well as damaged and aggregate-prone proteins. Autophagy has been shown to be involved in normal tissue remodeling, embryogenesis, in counteracting age-related physiologic changes, and in foreign antigen presentation to the immune system [11,15,16]. However, aberrant regulation of autophagy has also been associated with development of pathologies such as neurodegenerative disease and carcinogenesis. Massive autophagic vacuolization has been observed in some instances of cell death. [11,15,17,18]

### 2.3. Necrosis

In both type 1 and type 2 cell death, the remains of cells are disposed of by phagocytosis without an inflammatory response. This is in contrast to necrosis, the type 3 cell death, in which nonspecific and non-physiological stress, such as osmotic lysis, may induce diffusion of lytic enzymes and eventually cause cell destruction and a strong inflammation response. Morphologically, necrosis is characterized by an enlarged cell body (oncosis) that finally culminates in rupture of the plasma membrane and the dismantling of swollen organelles [9,11,19].

### 2.4. Necroptosis

While necrosis has been considered non-programmable and unregulated, another form of programmed cell death, necroptosis, has been characterized in recent years [19,20]. It is a special type of cell necrosis initiated by engagement of death receptors (TNF-R1) and requires the kinase activity of receptor-interacting protein 1 (RIP1; also known as RIPK1) and RIP3 (also known as RIPK3) [19,20]. In contrast to necrosis, necroptosis can be regulated by genetic, epigenetic, and pharmacological factors [20]. For example, pharmacological or genetic inactivation of caspases can cause cells to exhibit morphologies with either mixed necrotic and apoptotic features or complete necrosis [19]. Necroptosis participates in the pathogenesis of many diseases, including ischemic injury, neuro-degeneration and viral infection. Like necrosis, necroptosis has been considered harmful to the body because massive cellular disintegration may promote severe local inflammation which may, in turn, support tumor growth [21].

## 3. Cell Death Processes Activated in Prostate Cancers after ADT

While apoptosis has been well documented as a primary mechanism for prostate tumor regression after ADT, it is likely not the only type of cell death mechanism involved. Upon androgen withdrawal, autophagosomes, the hallmark of autophagy, have been observed in human prostate cancer cell lines [22,23]. Autophagy has been reported to have a protective role in prostate cancer cells but it may also induce autophagic cell death [9,22]. Since androgens have been reported to regulate blood flow to the prostate [24], ADT may lead to decreased blood flow to the prostate, resulting in a hypoxic environment with accompanying apoptosis of capillary endothelial cells in the tumor bed [24-26]. The apoptotic pathways in prostate cancer cells may subsequently be blocked by increased expression of hypoxia-inducible factor I (HIF-1) and this anoxic stress, induced by ADT, may render the cells unable to initiate an apoptotic cascade. The apoptotic process may then be eventually supplanted by necrosis, probably with necroptosis as the intermittent process. Hence different modalities of cell death including apoptosis, autophagy, necroptosis and necrosis may be induced by ADT, making it an effective therapy to treat locally advanced and metastatic androgen-dependent prostate cancers. However, prostate cancer cells eventually progress to an androgen-independent (AI) or castration-resistant state where conventional ADT is ineffective. When this occurs, the median survival of the patient decreases to ∼16–18 months [2,27,28]. Thus, a major obstacle for successfully treating prostate cancer and prolonging survival is progression to castration resistance with its attendant failure to activate cell death mechanisms.

While activation of apoptosis is considered beneficial for treating prostate cancer, activation of autophagy can have the opposite effect. Indeed, suppression of the autophagic process pharmacologically or by knocking down Beclin 1, a molecule that is known to be necessary for autophagy and to regulate autophagic initiation by forming a complex with PI3K, results in increased apoptosis. Autophagy may serve to protect the cells from ADT induced cell apoptosis and thereby promote castration resistant prostate cancer (CRPC). If cells survive metabolic, genotoxic or anoxic stress, their capacity to initiate further apoptotic cascades may be blocked by endogenous inhibitors, including high concentrations of anti-apoptotic molecules such as hypoxia HIF-1 and Bcl-2, or through down regulation of pro-apoptotic molecules like Bad. As mentioned above, under these conditions apoptosis may be replaced by necrosis. While necrosis can result in shrinkage of the tumor, massive cellular disintegration may also promote severe local inflammation and support tumor growth [21]. The hazardous effects of the apoptotic to necrotic conversion with accompanying release of necrotic mediators that may promote cancer progression is supported by clinical observations in which the presence of necrosis is almost always correlated with poor prognosis. High-mobility group box1 protein (HMGB1) for example, is one such mediator which is released by necrotic but not apoptotic cells. Recently, HMGB1 has been shown to be secreted from prostate cancer cells and its over-expression is associated with prostate cancer progression [13,17].Though HMGB1 can regulate gene expression, it acts primarily as a cytokine to promote angiogenesis and extravascular migration of inflammatory cells. This initiates inflammatory reactions which may promote cancer growth [4,34]. Accordingly, necrosis may also be induced after ADT and signals released from necrotic cells such as HMGB1 may be important mediators for promoting the emergence of CRPC. Interestingly, HMGB1 itself is critical for the survival of cancer cells since knockdown of HMGB1 results in a caspase-3-dependent apoptosis [29]. In conclusion, it is thus conceivable that in order to make ADT more effective, suppression of autophagy, which is protective against cells death, and blocking survival signals mediated through necroptosis or necrosis may be necessary.

## 4. Mechanisms Underlying Progression of Prostate Cancers to Castration Resistance

### 4.1. Persistence of Active AR is Pivotal to the Progression of CRPC

Acquisition of the capacity for growth self-sufficiency and the evasion of cell death processes are considered to be critical hallmarks for the malignant progression of cancers [12]. In the majority of prostate cancers, formerly androgen-dependent cells acquire the ability to grow in an androgen-deprived environment and to evade apoptosis and other cell death processes which were previously active. We and others have shown that the AR remains active in most castration resistant prostate cancers (CRPCs) and that knocking down the AR in CRPC model systems can often cause complete regression of these tumors [30-32]. Amplification of the AR gene and over-expression of AR have also been observed in CRPC [33-36]. High levels of nuclear AR are found in most locally advanced CRPCs and in bone metastases. This is further supported by increased AR mRNA levels consistently observed with the progression to CRPC in gene microarray studies [30]. Overexpressed AR may thus enable cancer cells to respond to low levels of androgen and to transduce signals sufficient for cell growth and survival.

### 4.2. AR Mutations

AI growth and survival in CRPC can also be maintained by constitutively active mutant ARs [37,38]. The incidence of somatic mutation in the AR in prostate cancer samples occurs more frequently in tumor cells of distant metastases and recurrent prostate cancer following ADT compared to that seen in primary prostate cancers. Mutations in the AR may impart altered sensitivity and specificity. For example, point mutations in the AR gene identified in metastatic cells from patients and from AI prostate-cancer cell lines could be activated by progesterone and estrogen [37]. An AR gene harboring a gain-of-function mutation which is constitutively active can thus render prostate cancer cells independent of androgen and with a castration-resistant phenotype [37,38]. Moreover, in recent years, constitutively active AR splice variants lacking the ligand binding domain have been identified in AI prostate cancer cell lines and tissues, with the highest levels observed in late stage CRPC [39-44]. Targeting the ligand binding domain with anti-androgens or selective siRNA silencing of full length AR blocked the cell growth-promoting effects of constitutively active AR variants, indicating that the gain-of-function of such constitutively active AR splice variants still requires the full-length AR [40]. However, the fact that such AR variants are capable of conferring anchorage-independent growth *in vitro* and CRPC growth *in vivo* [40,43] and that they can regulate the expression of full-length AR and modulate AR activity [39,44] suggests their important roles for the progression of CRPC.

### 4.3. AR Coregulators

AR modulates transcription of androgen-responsive genes via recruitment of coregulators that influence a number of functional properties of the receptors, including ligand selectivity and DNA binding capacity [45-48]. There exist four main types of coregulators for AR: (1) molecular chaperones that coordinate AR maturation and movement, (2) histone modifiers, (3) coordinators of transcription and (4) DNA structural modifiers. Functionally, co-regulators interact with AR to either promote (co-activator) or inhibit (co-repressor) AR activity. While AR activity may be promoted when coactivator levels increase, it has been demonstrated that AR recruitment of corepressors is a key mediator of antagonist mediated inhibition of steroid receptors, suggesting that loss of corepressor expression and the subsequent release of the suppression of AR activity may facilitate the progression to CRPC [48].

### 4.4. Crosstalk of AR with other Cell Signaling Pathways

There are at least four prominent signaling pathways through which AR crosstalk may contribute to castration resistance: (i) the PI3K/Akt pathway, (ii) receptor tyrosine kinases, (iii) the p38 MAPK pathway, and (iv) the Wnt/β-catenin pathway.

#### 4.4.1. AR crosstalk with PI3K/Akt Pathway

Perhaps the most important pathway activated in CRPC progression is the phosphatidylinositol 3-kinase (PI3K)/Akt pathway. Overexpression of insulin-like growth factor 1 receptor (IGF-1R) has been observed in many prostate cancers and, in particular, it is upregulated *in vivo* during the progression to castration resistance [49,50]. Signaling through the upregulated IGF-1R results in enhanced autophosphorylation and tyrosine phosphorylation of IGF-IR substrates, which enables the recruitment of different effectors to induce potent activation of the PI3K/Akt pathway [51]. Activation of PI3K leads to phospho-activation of Akt and subsequent inhibitory phosphorylation of the pro-apoptotic molecule, Bad. Consequently, the anti-apoptotic capacity is amplified within the cancer cells [49,50,52]. In addition, loss of PTEN, a tumor suppressor gene encoding a phosphatase to suppress PI3K, provides another very important mechanism for progression to CRPC. Indeed, PTEN function is lost by gene deletion or mutation in more than 60% of prostate cancers, notably those with high Gleason scores and advanced pathological stage. By eliminating its negative regulation function, loss of PTEN will lead to activation of the PI3K/Akt pathway and activation of the anti-apoptotic signaling as mentioned above [53-56]. Direct evidence that loss of PTEN regulates cell survival is based on the observation that ectopic expression of PTEN in prostate cancer cells suppresses transcription of the anti-apoptotic molecule Bcl-2 via the cAMP response element-binding protein (CREB). Accordingly, loss of PTEN abrogates CREB-mediated suppression of Bcl-2 and thus may contribute to increased Bcl-2 levels in advanced CRPC [57]. Indeed, more than 80% of prostate cancer samples with increased Bcl-2 expression were also found to have a loss of PTEN. In addition to the anti-apoptotic or survival signals mediated through activated PI3K, signaling through activated PI3K regulates AR activities by inducing AR phosphorylation, altering AR transcription, and promoting AR translocation into the nucleus [58-60], all of which may contribute to progression to CRPC.

#### 4.4.2. AR Crosstalk with Receptor Tyrosine Kinases

Overexpression and amplification of human epidermal growth factor 2 (HER-2) occur in many cancers and are associated with poor prognosis [61-64]. Though it is well accepted that the HER-2 gene is not often amplified in primary prostate cancers [62,64-68], our observations together with others have demonstrated that overexpression of HER-2 proteins is associated with CRPC progression [62,69]. HER-2 forms heterodimers with other EGFR family members and these complexes become hypersensitive to activation by various ligands such as EGF, TGFα, neuregulins, *etc* [65,70]. Ligand binding to HER-2 initiates signaling cascades leading to potent activation of Ras/Raf/MAPK, which results in increased cell proliferation and decreased cell death [70-72]. Importantly, over-expressed HER-2 can dimerize with ERBB3 to transduce signals for stabilizing AR protein levels and optimize binding of AR to promoter/enhancer regions of androgen-regulated genes, thereby promoting AR activities [73]. Hence, overexpressed HER-2 is capable of conferring AI growth both *in vitro* and *in vivo* and promoting CRPC progression [61,72].

#### 4.4.3. AR Crosstalk with the p38 MAPK Pathway

Activation of the p38 MAPK pathway may be another mechanism for progression to CRPC. This is particularly true when prostate cancer cells are in a hypoxic environment. Many solid tumors have hypoxic regions which are associated with disease progression and treatment resistance [23]. Since androgens have been reported to be able to regulate blood flow to the prostate [24], ADT will lead to decreased blood flow resulting in a hypoxic environment and subsequent apoptosis of capillary endothelial cells [24-26]. Upon induction of hypoxia, p38 MAPK is activated and phosphorylates HSP27 which can then translocate into the nucleus to stabilize AR [74].

#### 4.4.4. AR Crosstalk with the Wnt/β-catenin Pathway

There are two types of Wnt/β-catenin signaling pathways: the canonical and non-canonical. In the canonical pathway, Wnt ligands bind members of the Frizzled family transmembrane receptors resulting in the stabilization of β-catenin, which translocates into the nucleus to form a transcriptional complex with T-cell factor (Tcf) and lymphoid enhancer factor (LEF) [75]. Expression of genes involved in cell proliferation and apoptosis, such as c-myc, AP-1 members, c-jun and fra-1 and cyclin D1, are subsequently initiated by this complex. In addition, β-catenin is able to promote AR activity by directly interacting with AR. Physically, β-catenin binds to the ligand binding domain (LBD) and *N*-terminus of the AR via the first six armadillo repeats and this interaction enhances AR-mediated transcription [76-79]. Deregulation of the Wnt/β-catenin pathway in advanced CRPC has been noted by many investigators [80-83]. Members of the canonical Wnt pathway, including Wnt-3a and Wnt-1 and β-catenin, have been shown to be up-regulated in metastatic prostate cancer and β-catenin and AR have been shown to co-localize in the nucleus in advanced prostate cancer samples. Interestingly, upon activation with a canonical Wnt ligand, AR can activate transcription of androgen responsive genes (e.g., PSA) in the absence of androgen and thereby support AI cell growth. This process is associated with formation of AR-β-catenin complexes at the PSA promoter in the nucleus [84,85]. At castration levels of androgen, unbound AR was also found to be recruited to TCF/LEF/β-catenin-regulated promoters of genes such as myc and cyclin D1 which are critical for cell growth and survival [85]. Expression of those genes may promote progression to CRPC.

### 4.5. De Novo Synthesis of Androgen

In most cases of CRPC, tumors resume their expression of multiple AR-regulated genes such as PSA, TMPRSS2:ERG fusion genes, *etc* [86,87], indicating the reactivation of AR. As discussed previously, elevated expression levels of AR, AR mutations that transduce weak androgen signals, high levels of constitutively active AR splice variants lacking ligand binding and increased expression of transcriptional coactivator proteins may contribute to the reactivation of AR. In addition, it has been observed that intraprostatic levels of androgens remain moderately high in CRPC, which is the result of *de novo* synthesis of testosterone and DHT from weak adrenal androgens or from cholesterol within CRPC cells [87-89]. The reactivation of AR by such de novo synthesized androgen may be another molecular mechanism for the progression of CRPC.

In conclusion, hyperactive AR signaling by any of the mechanisms discussed above is linked to cell survival and the progression of CRPC.

### 4.6. Prostate Cancer Stem/Progenitor Cells

It is believed that there may exist prostate cancer stem/progenitor cells which may arise from normal tissue stem cells [90]. These prostate cancer stem/progenitor cells are likely AR negative [90,91]. As discussed previously, ADT has been designed to eradicate cancer cells within a tumor by targeting the AR positive population. However, by eradicating androgen-dependent prostate cancer cells, ADT may actually promote disease progression by activating normally quiescent cancer stem cells to repopulate the tumor with AR negative, androgen-independent cells [90-92]. Although unproven, the repopulation of the prostate tumor with androgen-independent cancer cells may be a very important mechanism in progression to CRPC.

## 5. Conclusions

Cells die in at least four distinct manners including apoptosis, autophagy, necrosis and necroptosis. Extrinsic or intrinsic stimuli such as DNA damage, loss of cell cycle control, hypoxia and withdrawal of growth factors as well as numerous chemo- or radio- therapies may cause cells to die in one or more of these cell death modalities. As the most widely used therapy for locally advanced and/or metastatic prostate cancers, ADT has proven to be effective in inducing extensive cell death of cancer cells. However, prostate cancers progress to become refractory to castration-induced cell death. While epigenetic modulation or genetic mutation in the AR play important roles in CRPC progression, the persistence of a functional AR is also critical. The AR can function as an interface to crosstalk with various signaling pathways to provide signals sufficient enough for cell proliferation and survival in the absence of androgen. Hence, targeting the AR as well as downstream signaling molecules critical for progression to castration resistance may provide effective therapies to treat CRPC and increase patient survival.
